# Recent Progress in Advanced Electrode Materials for the Detection of 4-Nitrophenol and Its Derivatives for Environmental Monitoring

**DOI:** 10.3390/s26010306

**Published:** 2026-01-03

**Authors:** Shanmugam Vignesh, Chellakannu Rajkumar, Rohit Kumar Singh Gautam, Sanjeevamuthu Suganthi, Khursheed Ahmad, Tae Hwan Oh

**Affiliations:** 1 School of Chemical Engineering, Yeungnam University, Gyeongsan 38541, Republic of Korea; 2 Department of Mechanical Engineering, Teerthanker Mahaveer University, Moradabad 244001, India

**Keywords:** nitroaromatics, p-NP, 4-NP, electrochemical sensors, environmental monitoring

## Abstract

It is understood that 4-nitrophenol (4-NP) and its derivatives/isomers, such as m-NP and o-NP, are considered toxic nitroaromatic pollutants that pose health risks for human beings and have negative impacts on the environment. Therefore, monitoring of 4-NP is of particular importance to avoid the negative impacts of these environmental pollutants on aquatic life and human health. Electrochemical sensors have emerged as the most promising next-generation technology for the detection of environmental pollutants. The electrochemical method has been extensively used for the detection of 4-NP, p-NP, etc., which has delivered an interesting electrochemical performance. This review provides an overview of the advances in electrode modifiers designed for the electrochemical detection of 4-NP and its isomers. This review includes the use of carbon-based materials, metal oxides, metal sulfides, metal-organic-frameworks (MOFs), conducting polymers, MXenes, covalent organic frameworks (COF), and composites for the development of 4-NP electrochemical sensors. Various electrochemical techniques, such as differential pulse voltammetry, square wave voltammetry, linear sweep voltammetry, cyclic voltammetry (CV), electrochemical impedance spectroscopy, and amperometry, are discussed for the detection of 4-NP and other isomers.

## 1. Introduction

Environmental pollution is one of the major concerns of the present world [1,2]. There are various environmental pollutants such as nitroaromatics and phenolic compounds that come out from various industries [3,4]. In particular, nitroaromatic compounds, such as 4-nitrophenol (4-NP) and its derivatives (m-NP and o-NP), are widely used in dyes, pesticides, pharmaceuticals, and other industrial processes [5,6,7]. 4-NP and its isomers (o-NP and m-NP) show toxicity, persistence, and a bio-accumulative nature, which makes them priority environmental pollutants [8,9]. The long-term exposure to these environmental pollutants may affect human health and cause various diseases in humans [10]. In addition, these environmental pollutants may have negative impacts on aquatic life and be responsible for environmental pollution [11]. A previous study stated that the acceptable concentration of nitroaromatic compounds is a few μg/L in aquatic environments [2]. In another study [3], it was also mentioned that the tolerable limit of phenols in wastewater and drinking water is 1 ppm and 0.5 ppb, respectively. Therefore, the detection of 4-NP or its isomers is of great significance for environmental pollution control. It is known that conventional methods, such as high-performance liquid chromatography (HPLC) [12], gas chromatography–mass spectrometry (GC–MS) [13], fluorescence spectroscopy [14], and spectrophotometry [15], can be used for the monitoring of environmental pollutants. Unfortunately, these conventional methods have their own limitations: they require high-cost instrumentation, are time consuming, require trained operators, and have portability issues [16,17]. Therefore, the electrochemical method was employed as an alternative to conventional methods that offers several advantages, such as simple electrode fabrication, acceptable recovery in real samples, and high selectivity and sensitivity [18]. However, it was observed that bare electrodes, such as the glassy carbon electrode (GCE), could not catalyze the redox reactions for the detection of 4-NP due to their poor catalytic activity. Thus, the bare GCE needs to be modified with an electrocatalyst to enhance the redox reactions at the electrode surface for the detection of 4-NP. Electrocatalysts with high specific surface area, catalytic properties, and high electrical conductivity are considered promising electrode modifiers for electrochemical sensing applications [18,19,20]. Carbon-based materials offer several advantages, including high conductivity, a larger specific surface area, and structural properties [21]. Thus, carbon-based materials have been extensively used as electrocatalysts for the detection of 4-NP [22,23,24].

**Figure 1:**
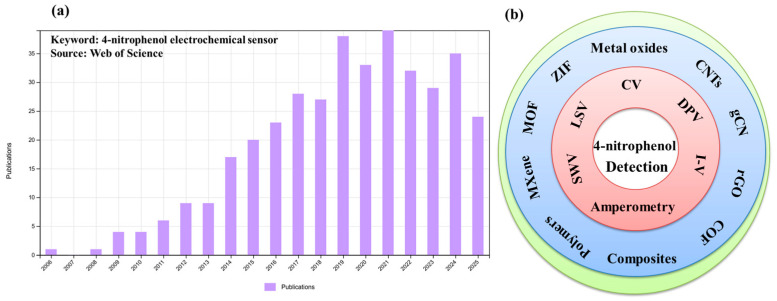
(**a**) Bar graph shows the previous publications on 4-NP electrochemical sensors (source: Web of Science). (**b**) Schematic graph shows different materials for 4-NP detection.

Although numerous reports are available in the past decades on the detection of 4-NP (Figure 1a), this review article summarizes recent progress (previous five years) in electrode materials for the construction of electrochemical sensors for the monitoring of 4-NP and its derivatives. Various electrode modifiers, such as carbon, metal-organic-frameworks (MOFs), covalent-organic-frameworks (COF), MXenes, metal oxides, etc., have been reviewed for the construction of 4-NP sensors (Figure 1b). The challenges and future perspectives have also been discussed. It is believed that the present review article may be beneficial for electrochemists who are working on the development of electrochemical sensors for the monitoring of environmental pollutants.

## 2. Progress in Electrochemical Detection

### 2.1. Electrochemical Sensors Based on Carbon-Based Materials

#### 2.1.1. Graphitic Carbon Nitride (gCN)-Based Materials

**Figure 2:**
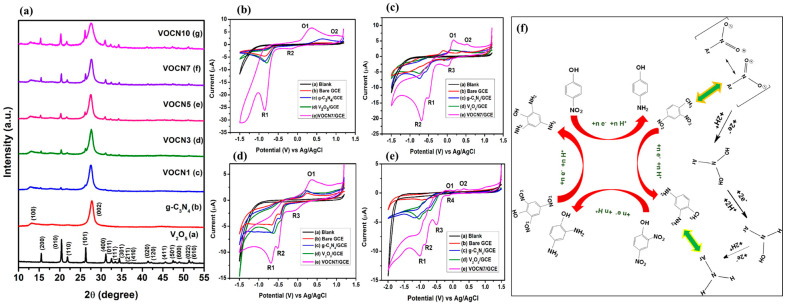
(**a**) XRD patterns of the prepared V_2_O_5_ (**a**), gCN (**b**), VOCN1 (**c**), VOCN3 (**d**), VOCN5 (**e**), VOCN7 (**f**), and VOCN10 (**g**). CV curves of blank solution, bare GCE, gCN/GCE, V_2_O_5_/GCE, and VOCN7/GCE in the presence of (**b**) 4-NP, (**c**) DNP, (**d**) DNT, and (**e**) TNP. (**f**) Probable reaction mechanism for nitroaromatics detection. Reproduced with permission [22].

Carbon-based materials have been extensively used as electrode modifiers for the construction of electrochemical sensors for nitroaromatic detection. In this regard, Jiang et al. [21] proposed that graphitic carbon nitride (gCN) may be a promising electrode modifier for the detection of 4-NP. However, the low conductivity of gCN needs to be improved to enhance the sensitivity of the 4-NP sensor. Therefore, the authors proposed a doping strategy that may improve the electrical conductivity and catalytic activity of gCN for the detection of 4-NP. The phenanthroline-doped gCN (gCN-PD) was synthesized using 1, 10-phenanthroline-5, 6-dione and urea as precursors. The X-ray diffraction (XRD), scanning electron microscopy (SEM), and energy dispersive X-ray spectroscopy (EDX) studies authenticated the formation of gCN-PD, with reliable phase purity and a sheet-like structure. It was also found that phenanthroline doping extended the π-conjugation process of gCN and generated defect sites in gCN. Thus, the prepared material exhibited improved conductivity, adsorption, and electrocatalytic behavior for 4-NP. Cyclic voltammetry (CV) and differential pulse voltammetry (DPV) were adopted as the sensing technique for 4-NP detection. The CN-PD-modified glassy carbon electrode (GCE) displayed a limit of detection (LOD) of 39 nM and broad linear range of 2.854 µM to 1117 µM, with reasonable recovery in real-life water samples. This work suggested that doped gCN materials exhibit better electrochemical properties compared to pristine gCN. The incorporation of metal oxides in gCN may also display synergistic interactions and enhance the properties of the resulting composite materials. Therefore, Sangamithirai et al. [22] prepared a vanadium oxide (V_2_O_5_)- and gCN-based composite (gCN/V_2_O_5_) using hydrothermal/sonochemical-assisted methods. The authors prepared a series of gCN/V_2_O_5_ materials (V_2_O_5_ = 1, 3, 5, 7, and 10 wt %) to optimize the electrochemical behavior of the proposed catalyst for the detection of 4-NP and other nitroaromatics, such as dinitrophenol (DNP), dinitrotoluene (DNT), and trinitrophenol (TNP). The XRD patterns of the prepared samples (V_2_O_5_, g-C_3_N_4_ = gCN, VOCN1, VOCN3, VOCN5, VOCN7, and VOCN10) are shown in Figure 2a, confirming the successful formation of the target materials. The XRD patterns also revealed the formation of composite materials with good purity. Furthermore, various electrodes were developed using gCN, V_2_O_5_, and VOCN7 as the electrode material. The CVs of the bare GCE, gCN/GCE, V_2_O_5_/GCE, and VOCN7/GCE in the presence of 4-NP, DNP, DNT, and TNP are shown in Figure 2b, Figure 2c, Figure 2d, and Figure 2e, respectively. In the case of 4-NP, one irreversible reduction peak (R_1_) and a pair of reversible oxidation (O_1_) and reduction (R_2_) peaks were observed. O_2_ is an irreversible oxidation process for the phenolic group. The R_1_ peak corresponds to the conversion of 4-NP to 4-hydroxyamino phenol. The O_1_ peak can be assigned to the oxidation of p-hydroxyl-amino phenol to 4-nitroso-phenol, and the R_2_ peak corresponds to the subsequent reversible reduction process. The reaction mechanism for nitroaromatic detection is shown in Figure 2f.

**Figure 3:**
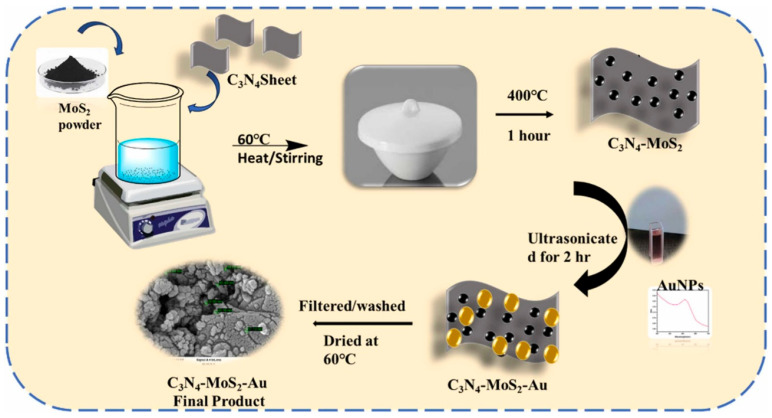
Schematic graph shows the formation of gCN/MoS_2_-Au. Reproduced with permission [26].

The investigations suggested that the 7 wt % V_2_O_5_-based composite with gCN exhibits a higher sensitivity for the detection of 4-NP and other derivatives. Square wave voltammetry (SWV) and amperometry techniques were also used to determine these nitroaromatics. For 4-NP detection, an LOD of 0.85 nM, a linear range of 1 nM to 100 µM, an acceptable selectivity, high reproducibility, and reusability were achieved under the optimized conditions. As mentioned above, gCN/V_2_O_5_ exhibits the lowest LOD value for 4-NP detection, which can be ascribed to the presence of synergistic effects, such as formation of a hetero-structure, surface morphology, and electrostatic interactions between the modified electrode and 4-NP. It is clear that redox-active V_2_O_5_ nanobelts with porous and N-rich gCN form a hetero-junction, which improves the interfacial charge transfer. The presence of porosity and the larger surface area of gCN may also enhance the adsorption of 4-NP at the electrode surface through π–π interactions. The presence of the V^5+^ state in V_2_O_5_ plays a vital role and facilitates electron transfer during redox reactions. The optimized gCN/V_2_O_5_ composite also shows low charge transfer resistance, thereby enhancing the redox reaction. Thus, gCN/V_2_O_5_ displayed an interesting sensing performance for 4-NP detection. It is also known that the microwave method has various advantages for the preparation of nanostructured materials. Therefore, a microwave-assisted preparation of a multifunctional hierarchical ternary composite of gCN with bismuth vanadate and silver carbonate (BiVO_4_/Ag_2_CO_3_) was also reported for electrochemical detection of 4-NP [23]. The electrochemical studies revealed that the proposed material has high activity for the detection of 4-NP, with a DL of 0.012 µM and sensitivity of 2.368 μA.mM^−1^·cm^−2^. The authors observed that the presence of synergism and electronic interactions between gCN and BiVO_4_/Ag_2_CO_3_ enhances the surface area and active sites of the fabricated electrode, which improves the sensitivity of the 4-NP sensor. gCN was also combined with cerium oxide/cadmium oxide (CeO_2_/CdO) under facile conditions [24]. The obtained hybrid composite was coated on a GCE surface, and its electrochemical performance for 4-NP detection was evaluated using linear sweep voltammetry (LSV) and CV. The kinetics and conductivity of the fabricated composite was determined using electrochemical impedance spectroscopy (EIS), which indicated the existence of high electrical conductivity in the electrode material. The authors also used nafion as a binder to enhance the adhesiveness of the electrode material to the GCE surface. It is worth mentioning that nafion significantly enhanced the adhesiveness of the catalyst to the electrode surface and improved the stability of the fabricated electrochemical sensors for long-term applications. However, the presence of nafion on the modified electrode surface reduced the conductivity of the catalyst, which can affect the sensitivity of the electrochemical sensors. Therefore, it is crucial to design and develop binder-free electrochemical sensors for practical applications. An LOD of 0.45 µM was achieved for 4-NP detection, with reasonably good recoveries in real samples. A high selectivity was also observed for 4-NP detection in the presence of various interfering substances, such as 4-methoxyphenol, bisphenol A, 2-NP, 4-aminophenol, and catechol. The presence of metal ions, such as K^+^, Ca^2+^, NO_3_^−^, Ag^+^, and Mg^2+^, with 500-fold concentration did not affect the selectivity of the proposed 4-NP sensor. Metal sulfides, such as molybdenum sulfide (MoS_2_), have interesting electrochemical properties, but the low conductivity of MoS_2_ is one of the major concerns. Therefore, MoS_2_ was combined with S-doped gCN to improve the electrochemically active sites and conductivity. The prepared material exhibited synergistic interactions, such as active sites, improved conductivity, and a layered structure that facilitated electron transfer and enhanced the sensitivity of the 4-NP sensor [25]. The MoS_2_/SgCN composite-modified GCE displayed high conductivity, which was authenticated by the EIS technique, and the fabricated 4-NP sensor delivered an LOD of 12.8 nM. The real-sample studies were performed using a standard spike method in water samples (concentration of 4-NP = 10, 15, and 20 µM). A recovery of 97.8% to 99.1% was observed in the real-water samples. In another study [26], a gCN/MoS_2_/Au composite was synthesized using a benign approach, as shown in Figure 3. The gCN/MoS_2_/Au was explored as a sensing material for the detection of 4-NP, DNP, and TNP using CV and LSV methods. The authors observed that the presence of Au with gCN/MoS_2_ not only improved the conductive nature of the modified electrode but also enhanced the catalytic properties for 4-NP detection. Therefore, the authors were able to obtain an LOD of 0.219 µM and a linear range of 0 to 1000 µM.

#### 2.1.2. Graphene-, Graphene Oxide (GO)-, and Reduced Graphene Oxide (rGO)-Based Materials

An inkjet printed-paper electrode (PPE) was also modified with a graphene–silver (Gr-Ag) composite, and its application for 4-NP sensing was carried out using electrochemical techniques [27]. The optimized conditions indicated that the proposed electrode material may deliver an LOD of 2.7 µM, with a wide linear range of 3.125 to 100 µM. This type of electrode may be used for environmental monitoring applications for the detection of pollutants. Au NPs/rGO and biomass-derived activated carbon (AC) were also used for the construction of a 4-NP sensor [28]. The AuNPs/rGO/AC-modified GCE displayed an LOD of 0.36 µM, a sensitivity of 0.03436 μA/µM, and high selectivity for 4-NP detection in the presence of various interfering substances. A graphene oxide (GO)/cellulose nanofibril composite (CNF)-modified carbon paste electrode (CPE) was also explored as an electrocatalyst for the detection of p-NP using the DPV and LSV techniques, whereas the conductivity of the prepared material GO/CNF was evaluated using the EIS technique [29]. An LOD of 0.8 nM, selectivity, and accuracy for real samples suggested the potential of GO/CNF/CPE for the monitoring of p-NP in environmental samples.

**Figure 4:**
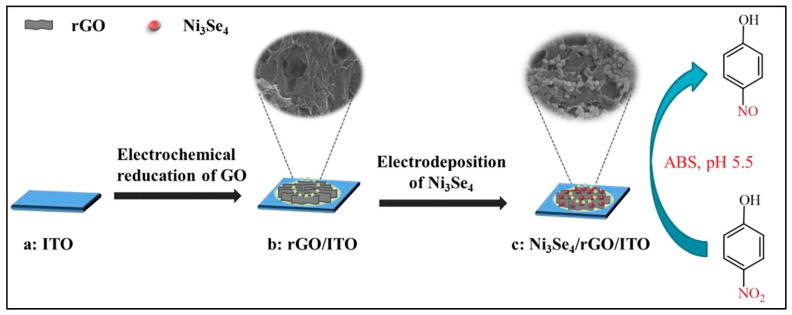
Schematic picture shows the fabrication of Ni_3_Se_4_/rGO/ITO for electrochemical sensing. Reproduced with permission [33].

In another study [30], a Gr-polyarginine composite was deposited on a screen printed electrode (SPE). This constructed electrode exhibited improved hydrogen bonding and ionic interactions. The synergism enhanced the electrochemical activity of the constructed electrode for the monitoring of p-NP in environmental samples. A novel composite of rGO, MoS_2_, and iron oxide (Fe_3_O_4_) was also designed and prepared for electrochemical applications [31]. The rGO-MoS_2_/Fe_3_O_4_ composite was characterized by Raman spectroscopy, which indicated an increase in D band intensity and suggested the introduction of non-graphitic defects in the prepared composite. The DPV studies displayed an LOD of 0.8 µM and a sensitivity of 0.71 μA.μM^−1^·cm^−2^ for p-NP detection. The rGO was also combined with MnO_2_ and deposited on the surface of a conductive fabric using facile conditions [32]. The fabricated rGO/MnO_2_/CF electrode delivered an LOD of 55.9 µM, a recovery of 99.9% in real samples, and selectivity for the detection of 4-NP. The conductive nature of rGO and catalytic behavior of MnO_2_ enhance the detection of 4-NP. Indium tin oxide (ITO) was modified with nickel selenide (Ni_3_Se_4_)/rGO for the sensing of 4-NP [33], as shown in Figure 4.

The Ni_3_Se_4_/rGO/ITO electrode showed an LOD of 17.1 nM and two linear ranges of 0.05 to 5 µM and 5 to 200 µM, with high reproducibility and an acceptable stability of 20 days. The template-free synthesis of zinc vanadate (ZV) microflowers was also reported to form the hybrid composite of ZV/rGO [34]. The presence of rGO offers several benefits, such as high surface area, high conductivity, and active sites, which enhance the detection of 4-NP. Therefore, it was observed that ZV/rGO-modified GCE can deliver an LOD of 0.009 µM and a linear range of 0.01 to 1955 µM. In another previous study [35], a Pd-graphene composite was prepared using histidine-functionalized graphene quantum dots (His-GQDs). Furthermore, a switchable thermal sensor was developed using Pd-His-GQD-G and poly (N-isopropylacrylamide) (PNIPAM). The fabricated sensor displayed an LOD of 0.1 µM and a linear range of 0.5 to 250 µM for the detection of 4-NP.

#### 2.1.3. Carbon Nanotube (CNT)-Based Materials

A flexible and wearable electrochemical sensor was also fabricated using a stencil printing technique [36]. Multi-walled carbon nanotubes (MWCNTs)/poly(3,4-ethylenedioxythiophene):poly(styrenesulfonate) (PEDOT:PSS) was employed as an electrode modifier. The proposed sensor displayed reasonably good sensing of 4-NP in real samples. This work offers several advantages, such as a low LOD, wide linear ranges, and selectivity for 4-NP and TNP using SWV.

A neodymium vanadate (NdV)/hydroxylated MWCNTs (NdV/MWCNTs-OH) composite was synthesized by an ultrasonic-assisted method [37]. This material was coated on a GCE surface, and its electrochemical performance for p-NP detection was checked by electrochemical techniques. The presence of synergism (high conductivity of MWCNTs-OH, catalytic behavior of NdV, and abundant active sites) between the MWCNTs-OH and NdV improved the charge transfer kinetics that enable the efficient detection of p-NP. The detection of p-NP in river water and industrial wastewater also offers the potential for application in environmental monitoring. MWCNTs-COOH were also functionalized with polyethylenimine (PEI) and coated on a GCE surface [38]. The PEI-MWCNTs-COOH/GCE was employed as an electrochemical sensor for the detection of p-NP and o-NP. The authors adopted the second derivative linear sweep voltammetry (SDLSV) technique for the detection of p-NP and o-NP. The synergy effects enhanced the interactions between MWCNTs-COOH and PEI and improved the selectivity and sensitivity of the fabricated electrode for p-NP and o-NP. The authors achieved an LOD of 0.6 µM and 0.04 µM and a linear range of 2 to 100 µM and 0.2 to 100 µM for the sensing of o-NP and p-NP, respectively.

A polyimide integrated with MWCNTs (PI-MWCNT) was also employed as a sensing material for the simultaneous detection of three NP isomers [39]. The DPV-based studies revealed that the proposed material has electrochemical activities for the monitoring of 2-NP, 3-NP, and 4-NP in terms of a linear range of 1 to 300 µM, 0.25 to 250 µM, and 0.25 to 400 µM for 2-NP, 3-NP, and 4-NP, respectively. The authors also obtained LODs of 0.50 µM for 2-NP and 3-NP and 0.64 µM for 4-NP using the DPV technique. In another study [40], a mercury-doped yttrium oxide intercalated functional MWCNTs (MWCNTs@Hg/YO) composite was also explored as a 4-NP sensing material. The GCE surface was modified with MWCNTs@Hg/YO as the catalyst and nafion as the binder. A sensitivity of 6.233 μA.μM^−1^·cm^−2^, a linear range of 0.01 to 23 µM, and an LOD of 0.196 µM were obtained for 4-NP detection under the optimized conditions. A novel carboxylated MWCNTs- and carboxylated GO-based electrode (f-MWCNTs/f-GO/GCE) was also developed for the detection of 4-NP [41]. The authors found that the fabricated electrode has remarkable conductivity and catalytic activity for the electrochemical detection of 4-NP using an amperometry technique. Therefore, an interesting LOD of 5.4 nM and a wide linear range of 0.018 to 700 µM were obtained.

#### 2.1.4. Other Carbon-Based Materials

A molecularly imprinted polymer (MIP) integrated platinum (Pt) single atom decorated porous hollow carbon polyhedron-based disposable electrochemical sensor was also reported for the detection of phenolic pollutants [42]. The reported sensor exhibits good electrochemical performance, but selectivity may be a major concern for real-time monitoring of targeted analytes in the presence of various isomers of 4-NP.

In another work [43], a carbon molecular sieve (CMS) was prepared by pyrolysis of organic molecules with an inorganic molecular sieve. The obtained CMS was coated on a GCE surface, and electrochemical studies revealed that it has the potential to detect p-NP with an LOD of 0.2 µM and a linear range of 2 to 1000 µM. A spherical onion-like carbon (OLC) electrode material was also utilized as a sensing material for the construction of a 4-NP electrochemical sensor [44]. The OLC/GCE displayed LOD of 3.74 nM and linear range of 0.05 to 120 µM. This performance is relative better compared to the CNTs/GCE under the similar conditions. The OLC/GCE also showed long-term stability and selectivity for 4-NP detection. This may be attributed to the presence of a porous spherical onion-shaped structure of carbon.

**Figure 5:**
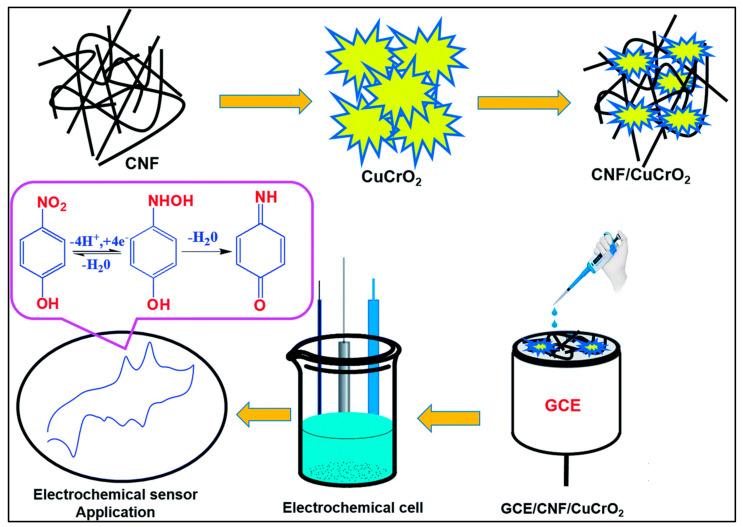
Schematic illustration of the fabrication of GCE/CNF/CuCrO_2_-based electrochemical sensor. Reproduced with permission [47].

Macroporous carbon (MPC) functionalized with triazine-bridged covalent organic polycalix [4] arenes (CalCOP) was also coated on a GCE for the electrochemical detection of p-NP, m-NP, and o-NP [45]. The constructed CalCOP-MPC-based electrode was capable of detecting p-NP and its isomers in environmental samples. LODs of 0.383 μM, 0.122 μM, and 0.212 μM were obtained for the detection of o-NP, m-NP, and p-NP, respectively. Nitrogen-doped mesoporous carbon (NCS) was also modified with nickel (Ni) for the development of 4-NP electrochemical sensors [46]. The prepared Ni/NCS was dispersed in polyethyleneimine (PEI) to form an ink, which was further deposited on the GCE surface. The NCS/PEI/GCE exhibited synergistic effects, such as high electrical conductivity, porosity, high surface area, electrocatalytic activity, and active sites. Therefore, a sensitivity of 1.465 μA.μM^−1^·cm^−2^, an LOD of 4 nM, and wide linear ranges of 0.06 to 10 µM and 10 to 100 µM were observed for 4-NP detection. The prepared electrode also showed a good selective nature for 4-NP sensing in the presence of interfering compounds. In another study [47], a carbon nanofiber/copper chromium dioxide (CNF/CuCrO_2_) composite was prepared using a hydrothermal-assisted synthetic method. The fabrication electrode is described in Figure 5.

It was observed that the CNF/CuCrO_2_-modified GCE has a higher current response for 4-NP detection compared to the bare electrode. The current response also increases with increasing 4-NP concentration. This 4-NP sensor also displayed high stability, reproducibility, cost-effectiveness, repeatability, and selectivity. It is understood from the above-mentioned discussion that electrochemical sensing of 4-NP involves a redox mechanism in which multi-step electron and proton transfer processes take place. The -NO_2_ group is reduced to hydroxyl-amine and further transformed to nitroso and amino intermediates at the surface of the modified electrodes. Thus, the selection of electrode modifiers is related to their ability to enhance and facilitate the electron transfer and provide active sites to improve the adsorption of 4-NP molecules. It is observed from the above-summarized reports that carbon-based materials offer several advantages, such as high conductivity, porosity, defects, larger surface area, and active sites. The incorporation of carbonaceous materials with metal oxides, polymers, or other materials demonstrated enhanced electron transfer and high sensitivity for 4-NP detection. Thus, it is suggested that carbon-based materials are efficient electrode modifiers for the development of 4-NP sensors. The electrochemical performance of the carbon-based materials is summarized in Table 1.

#### 2.1.5. Electrochemical Reaction Mechanism

In general, a working electrode (catalyst-modified GCE or SPE) plays a vital role in the electrochemical detection of the targeted analyte. The catalyst present on the GCE surface enhances the redox reactions by facilitating electron transfer. Initially, the modified electrode gets activated in the presence of water containing phosphate-buffered saline (PBS) solution. Shahzad et al. [24] proposed that the attachment of the oxygen may activate the surface of the modified GCE. Furthermore, 4-NP forms unstable intermediates via reactions with ionic oxygen. It was also stated that oxygen is chemically absorbed. The oxygen is transformed to O^2−^ and O^−^ by accepting electrons (e^−^) from gCN@CeO_2_/CdO/nafion/GCE, as shown in the reactions given below:

e^−^ (gCN@CeO_2_/CdO/nafion/GCE) + O_2_ → O^2−^ (1)

e^−^ (gCN@CeO_2_/CdO/nafion/GCE) + O^2−^ → 2O^−^ (2)

**Figure 6:**
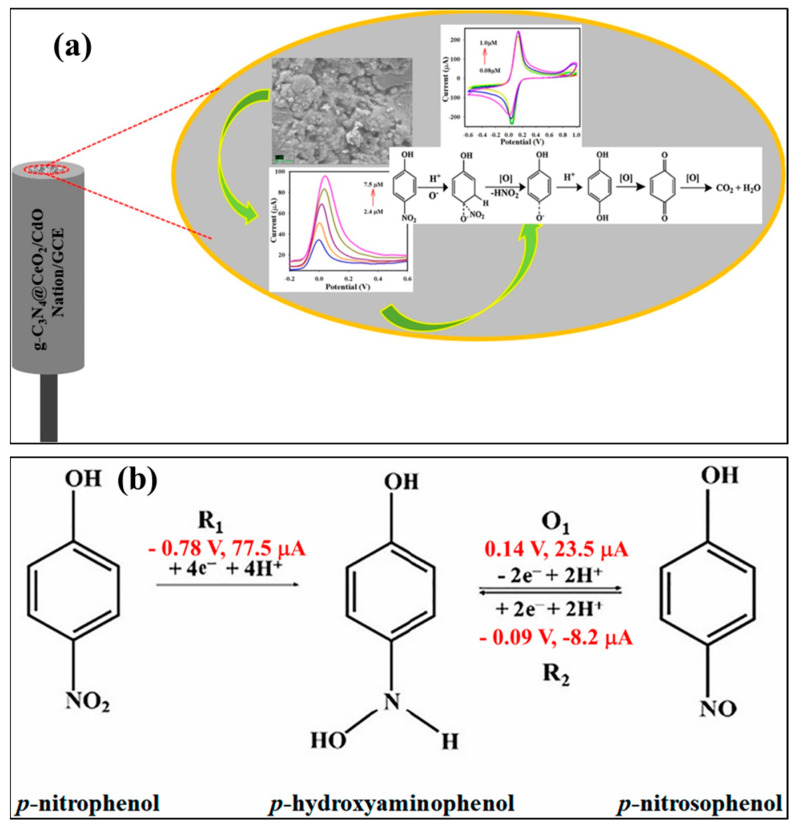
(**a**) Schematic representation of the electrochemical detection of 4-NP and its mechanism. (**b**) Reaction mechanism for p-NP detection. Reproduced with permission [24,31].

The electrons in the valence band of the gCN@CeO_2_/CdO/nafion composite are excited and transferred to their conduction band on applying an external potential. The hole remains in the valence band of the gCN@CeO_2_/CdO/nafion composite. The electrons from the conduction band of gCN are transferred to the conduction band of CeO_2_ and CdO, whereas holes migrate in the opposite direction. The transferred electrons participate in the electrochemical detection of 4-NP. The detailed mechanism for 4-NP detection is illustrated in Figure 6a. Kalia et al. [31] also proposed a mechanism for the detection of p-NP at the surface of an rGO-MoS_2_/Fe_3_O_4_-modified electrode. The reaction mechanism for p-NP detection is described in Figure 6b. It could be understood that the first step involves the transformation of p-NP to p-hydroxyaminophenol at a potential value of −0.78 V. The p-hydroxyaminophenol is oxidized to form p-nitrosophenol at a potential value of 0.14 V. The p-NP is transformed to p-hydroxyaminophenol at −0.09 V, and this process is reversible. However, the transformation of p-NP to p-hydroxaminophenol is an irreversible process.

Shahzad et al. [40] also proposed a sensing mechanism for the detection of 4-NP by an MWCNT@Hg/YO-modified electrode. The authors proposed that 4-NP is irreversibly reduced to 4-hydroxyaminophenol via four electrons and four protons at a potential of around 0 V. The fast electron transfer process takes place by the larger surface area and conductivity of MWCNTs, and the reaction is catalyzed by the Hg/YO composite. The 4-hydroxyaminophenol intermediate is transformed to 4-nitrosphenol by two electrons and two protons, and this process is reversible.

### 2.2. Metal Oxide-Based Electrochemical Sensors

#### 2.2.1. Metal Oxides, Doped Metal Oxides, and Composites

Cobalt oxide nanoparticles (CoO NPs) were prepared by a solution-processed method [48]. The prepared CoO NPs were deposited on a GCE surface, and the constructed CoO/GCE was explored as a 4-NP sensor. This sensor offers several advantages, such as high stability, reproducibility, and selectivity for 4-NP detection.

The microwave-assisted synthesis of silver oxide (Ag_2_O)/zinc oxide (ZnO) nanocones was also reported for the construction of a 4-NP sensor [49]. The presence of catalytic properties of ZnO and acceptable conductivity of Ag_2_O enhanced the sensitivity of the fabricated electrode for 4-NP sensing. Thus, an LOD of 23 nM and a sensitivity of 1.6 µA.µM^−1^·cm^−2^ were obtained for 4-NP monitoring.

**Figure 7:**
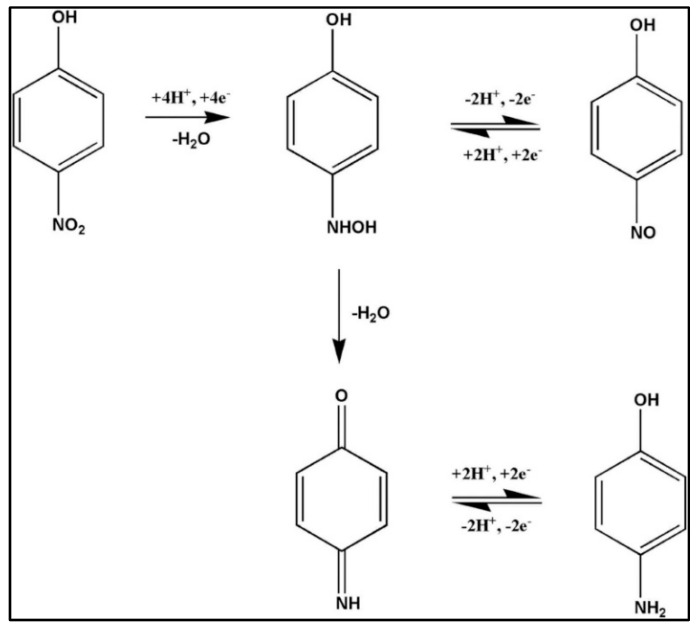
Possible sensing mechanism for 4-NP detection. Reproduced with permission [52].

Layered double hydroxide (LDH) materials offer a unique structure and surface area, which are beneficial for electrochemical reactions. α-Fe_2_O_3_@MgAl-CO_3_ LDH was deposited on the surface of a CPE (carbon paste electrode) [50]. The electrode material was prepared by a co-precipitation method. The α-Fe_2_O_3_@MgAl-CO_3_ LDH-modified CPE demonstrated an LOD of 4.98 µM and reasonable sensitivity using a voltammetric method. The spherical-shaped Ag_2_O NP, which has a particle size of 80 to 90 nm, was prepared by a microwave-assisted method [51]. The prepared materials were used as electrocatalysts for the monitoring of 4-nitrotoluene (4-NT) using LSV and CV methods. The proposed sensor was capable of exhibiting an LOD of 62.3 nM and linear ranges of 0.6 to 5.9 µM and 37 to 175 µM, with high sensitivity of 15.33 μA.μM^−1^·cm^−2^. The Ag_2_O NP/Au electrode also displayed reasonable reproducibility, selectivity, and reusability, but the presence of the Au electrode increases its cost. Therefore, it is clear that Au-electrode-free sensors would be beneficial for practical applications. Copper bismuth oxide (CuBi_2_O_4_), which is a ternary metal oxide, was prepared using a hydrothermal method [52]. The prepared CuBi_2_O_4_ has a nanorod (NR)-like surface structure and was drop casted on the electrode surface. 4-NP was used as the sensing target analyte, and electrochemical investigations revealed high sensitivity of 56.16 μA.μM^−1^·cm^−2^ and an LOD of 0.61 µM. The possible sensing mechanism for 4-NP detection is described in Figure 7.

Ho et al. [53] prepared a Ag-incorporated 3D flower-like porous Fe_3_O_4_ material using a quasi-reversible soft-template-assisted approach. The DPV analysis indicated that the fabricated electrode was highly sensitive towards 4-NP and delivered an LOD of 0.093 µM and a linear range of 1 to 15 µM. The presence of synergy between Ag NPs and flower-like Fe_3_O_4_ improved the catalytic activity of the electrode for the monitoring of 4-NP. The Fe_3_O_4_@Fe-BTC composite is also an interesting material for the detection of 4-NP using electrochemical technology [54]. The highly porous nature of Fe-BTC and synergistic effects of the electrode material facilitate electron transfer and enhance detection of 4-NP. Dildar et al. [55] adopted a wet chemical method for the synthesis of a zirconium oxide/neodymium oxide (ZrO_2_/Nd_2_O_3_) nanorod composite. PEDOT:PSS was also included as a conductive adhesive binder. The ZrO_2_/Nd_2_O_3_/PEDOT:PSS/GCE was found to be an attractive electrochemical sensor for the monitoring of p-NP. Samarium oxide (Sm_2_O_3_) may be utilized as an electrode material for the monitoring of various environmental pollutants [56]. Sm_2_O_3_ has various advantages, such as high electrical conductivity, electron transport properties, and thermal stability. Sm_2_O_3_ was coated on a Au electrode, and its electrochemical activity was evaluated for the monitoring of p-NP. This electrode was efficient and sensitive for the selective detection of p-NP. This sensor delivered an LOD of 0.50332 µM. Cerium vanadate (CeVO_4_) was prepared using a hydrothermal method and explored as a sensing material for the simultaneous detection of p-NP and TCP (trichlorophenol) [57]. The CeVO_4_-modified GCE exhibited an LOD of 0.091 µM and selectivity (interfering species were Mg^2+^, K^+^, Sr^2+^, citric acid, ascorbic acid, phenol, urea, and resorcinol) for p-NP detection using the SWV technique. Co_3_O_4_ doped with S was synthesized using a hydrothermal method and characterized by various sophisticated techniques [58]. The presence of a higher ratio of Co^2+^/Co^3+^ and increased oxygen vacancies in the prepared S-Co_3_O_4_ improved the electrochemical detection of p-NP. This electrode was found to be selective, sensitive, and stable for the monitoring of p-NP. In another previous study [59], a hydrothermal method was used for the synthesis of a Y@SnO_2_-ZnO composite for the detection of 2-NP. The Y@SnO_2_-ZnO/nafion/GCE delivered an LOD of 1.388 μM via the LSV technique. This electrode may be explored for the monitoring of 2-NP in environmental and healthcare applications. Amorphous MoO_X_ was successfully obtained using a one-step electrodeposition method [60]. The MoO_X_-based electrode exhibited high selectivity, stability, a sensitivity of 0.5266 μA.μM^−1^, and a low LOD of 0.0196 μM for p-NP detection.

#### 2.2.2. Metal Oxide Composites with Carbon-Based Materials

**Figure 8:**
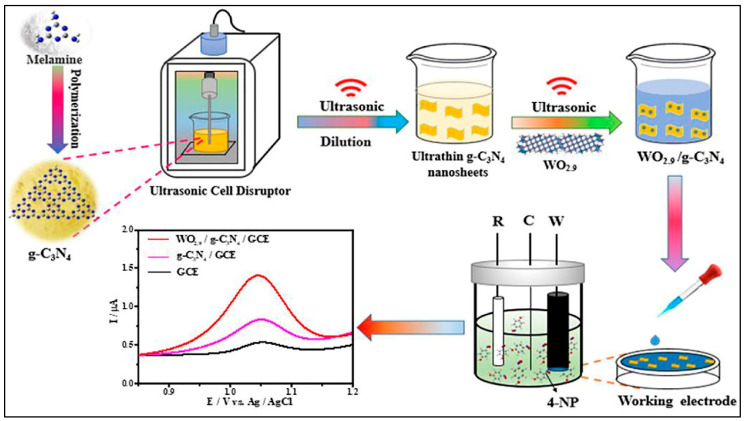
Schematic representation of the material preparation and electrode fabrication. Reproduced with permission [62].

In another study [61], a novel urushiol template-based solvothermal method was used for the synthesis of N-doped Gr (3D-Gr)/Mn-doped Fe_3_O_4_ NPs (Mn-Fe_3_O_4_/3D-Gr). The high surface area, porous nature, and active sites of the Mn-Fe_3_O_4_/3D-Gr improved the detection of 4-NP. Therefore, an LOD of 19 nM and an acceptable recovery of 98.38 to 100.41% in water samples were obtained for 4-NP detection. An oxygen vacancies (Vo) engineered WO_2_._9_/gCN composite was explored for the monitoring of 4-NP [62]. The material preparation and electrode fabrication are described in Figure 8.

The authors found that Vo improved the charge carrier density, created a built-in electric field, and enhanced adsorption energy at the interface of the hetero-junction. The proposed sensor displayed acceptable recovery and selectivity for 4-NP detection. In another report [63], nickel ferrite (NiFe_2_O_4_) NPs were incorporated with rGO, and the rGO amount was tuned in the wt % of 5, 10, 15, and 20%. The hydrothermally prepared above-mentioned materials were used as sensing material for p-NP detection using square wave anodic stripping voltammetry (SWASV). The authors found that the 10 wt %-based composite (NiFe_2_O_4_-rGO10/GCE) has higher electrochemical properties and catalytic activities for p-NP sensing. This proposed sensor has several advantages, such as a one-pot synthetic approach, cost-effectiveness, a wide linear range, a low LOD, and satisfactory recovery in real samples. Therefore, it can be stated that the proposed p-NP sensor can be used for practical applications. An L-cysteine-functionalized Nd_2_O_3_/rGO composite was employed as a sensing layer for the monitoring of o-NP and p-NP [64]. SWV, EID, and CV were used to evaluate the electrochemical sensing behavior of the L-cysteine-functionalized Nd_2_O_3_/rGO composite-modified electrode. The LOD of 0.01 µM and 0.02 µM were obtained for o-NP and p-NP, respectively, with reasonable selectivity. Wei et al. [65] prepared a zinc ferrite (ZnFe_2_O_4_)/polyaniline (PANI)@rGO aerogel composite using simple efforts. The constructed p-NP sensor exhibited a sensitivity of 36.898 mA.mM^−1^·cm^−2^ and a linear range of 1 to 100 µM for the detection of p-NP. The authors used tap water real samples and achieved acceptable recovery of p-NP. The presence of the 3D rGO aerogel framework and enhanced conductivity of the composite facilitated electron transfer. Therefore, an improved electrochemical performance of the fabricated p-NP sensor was observed. An rGO aerogel-decorated Pd NPs composite (rGO-A/Pd NPs) was prepared, and a RuO_2_-IrO_2_-Ti-rGO-A/Pd NPs electrode was developed for the monitoring of p-NP using an electrochemical method [66]. Although this sensor exhibited acceptable performance, its cost is higher due to the involvement of precious metals. Therefore, precious-metal-free electrodes need to be designed and developed for the monitoring of p-NP. Katowah et al. [67] adopted an in situ chemical oxidation approach for the preparation of a coconut-shell-activated carbon (CSAC)/strontium stannate (SrSnO_3_) composite with polymers such as polypyrrole (PPy), polycarbazole (PCz), and polyindole (Pin) to form CSAC/SrSnO_3_/PPy, CSAC/SrSnO_3_/PCz, and CSAC/SrSnO_3_/Pin, respectively. These prepared electrode materials were coated on a GCE surface, and electrochemical studies revealed that the CSAC/SrSnO_3_/PPy-based electrode had higher sensitivity for 4-NP detection. Thus, an LOD of 0.015 nM, a linear range of 1 nM to 10 mM, and sensitivity of 6.329 µA.mM^−1^·cm^−2^ were achieved. Titanium oxide-decorated carbon dots (TiO_2_-CDs) were prepared using a benign approach [68]. This prepared composite showed improved surface area and conductivity; therefore, the constructed electrochemical sensor was capable of displaying an LOD of 0.12 µM for 4-NP detection. A Ni@CuO/rGO-modified Pt electrode (PtE) was also capable of detecting 4-NP and exhibited an LOD of 0.0054 µM and a promising recovery rate for 4-NP in river and tap water samples [69]. Zhang et al. [70] prepared a ZnCo(OH)F-derived ZnCo_2_O_4_/MWCNTs composite for the sensing of 4-NP. This electrode was highly selective for the detection of 4-NP and displayed an LOD of 0.026 µM and a real-sample recovery of 98.79 to 100.47%. It is understood that the utilization of agro-waste is of great importance; therefore, CeO_2_@CDs were obtained using agro-waste [71]. The obtained material has a hollow nanospherical-shaped morphology. This material was used for dual application, including electrochemical sensing of 4-NP. A sensitivity of 43.4 μA/μM·cm^2^ and an LOD of 0.94 µM were obtained under the optimized conditions. It is understood that the electrochemical detection of 4-NP and its isomers involves redox reactions. Therefore, electrode modifiers should have high electrical conductivity to facilitate the redox reactions at the modified electrode surface. Metal oxides offer several advantages, such as stability, active sites, conductivity, and catalytic properties. The nanostructured metal oxide-based composites with conductive materials demonstrate synergistic interactions and improved conductive properties, which may accelerate electron transfer and enhance the sensitivity of the fabricated electrochemical sensors. Thus, as can be seen in Table 2, composite materials exhibit improved electrochemical performance for the detection of 4-NP and its isomers. As per the above discussion on metal oxide-based materials, it is observed that metal oxides, such as CoO, Fe_3_O_4_, ZnO, and CuBi_2_O_4_, have shown great promise for the construction of 4-NP sensors. However, it is understood that pristine metal oxides suffer from poor conductivity and a slow electron transfer process, which significantly affects the sensitivity of 4-NP sensors. The combination of metal oxides with carbon materials such as rGO demonstrates enhanced conductivity, low charge transfer resistance, and improved electron transfer. But some limitations still exist, such as the high temperature needed for metal oxide synthesis and gCN preparation. The electrochemical performance of the metal oxide-based electrode materials is summarized in Table 2.

#### 2.2.3. Sensing Mechanism

Dildar et al. [55] reported the sensing mechanism for the detection of p-NP using ZrO_2_-Nd_2_O_3_ NRs/PEDOT:PSS/GCE. The authors proposed that the porous morphology of the proposed composite plays a vital role and enables the chemical adsorption reaction with dissolved oxygen in the presence of PBS. The chemisorption leads to the acquisition of electrons from the conduction band of the composite material (ZrO_2_-Nd_2_O_3_ NRs), and O^2−^ and O^−^ are formed as shown in the reactions given below:

e^−^ (ZrO_2_-Nd_2_O_3_ NRs/GCE) + O_2_ → O^2−^ (3)

e^−^ (ZrO_2_-Nd_2_O_3_ NRs/GCE) + O^2−^ → 2O^−^ (4)

Furthermore, free hydroxyl radicals form due to the presence of surface-adsorbed water, as shown in the reaction given below:

e^−^ + O_2_ → O^2−^ + e^−^ → 2O^−^ + H_2_O → H_2_O_2_ → ***^•^***OH (5)

**Figure 9:**
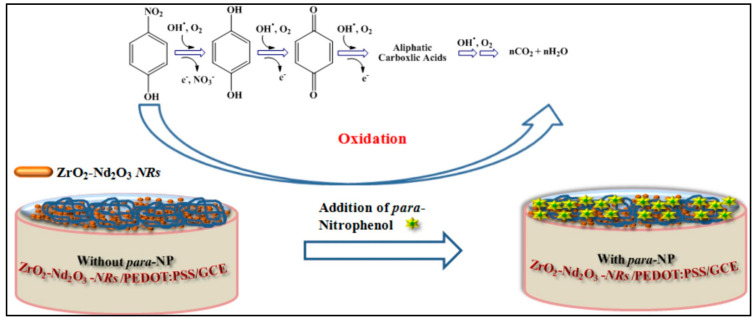
Schematic illustration of the sensing mechanism for p-NP detection. Reproducced with permission [55].

The p-NP is further electrohydrolyzed through these free hydroxyl radicals and forms hydroquinones with the release of nitrate ions and electrons. The authors also proposed that hydroxyl radicals are present in an abundant amount, and hydroquinones are oxidized to benzoquinones, which are further oxidized to aliphatic carboxylic acid and yield CO_2_ and H_2_O. The sensing mechanism for p-NP detection is described in Figure 9.

### 2.3. MOF/COF/ZIF/MXene-Based Electrochemical Sensors

Metal-organic-frameworks (MOFs) are high surface materials with high porosity. A novel Mn-based MOF was combined with rGO to modify the active surface of a GCE [72]. The modified electrode showed synergy effects, such as high surface area, interfacial interactions, high electrical conductivity, active sites, and catalytic activity, which may be beneficial for better electron transportation. Thus, the sensitivity of the constructed sensor can be enhanced for the detection of p-NP and its isomers, such as m-NP and o-NP. The obtained results exhibited LODs of 0.066 µM, 0.072 µM, and 0.078 µM and linear ranges of 0. 5 µM to 150 µM, 0.5 µM to 140 µM, and 0.5 µM to 180 µM for the sensing of p-NP, m-NP, and o-NP, respectively. Another research study showed the in situ preparation of a Cu-Pic coordination polymer (Cu-Pic CP) and a Cu-BTC MOF [73]. Furthermore, the prepared Cu-BTC MOF and Cu-Pic CP were used as a sensing material to modify the CPE. The Cu-Pic-modified electrode exhibited an LOD of 90 nM and a linear range of 1.2 µM to 70 µM for 4-NP detection. In contrast, the Cu-BTC-modified CPE displayed an LOD of 40 nM and linear ranges of 0.7 µM to 10 µM and 10 µM to 100 µM for 4-NP sensing. It was also found that both modified CPEs are selective and reproducible for the sensing of 4-NP. These sensors can be used to monitor the presence of 4-NP in environmental samples.

A copper-based MOF (Cu-MOF) was also incorporated with N-doped GO by a solvothermal method [74]. The Cu-MOF/N-GO was characterized by various techniques, such as EDX, Brunauer–Emmett–Teller (BET) analysis, SEM, high-resolution transmission electron microscopy (HRTEM), thermogravimetric analysis (TGA), XRD, etc., which confirmed the formation of the Cu-MOF/N-GO composite. The prepared material was deposited on an SPCE surface and used as a 4-NP sensor by employing the DPV technique. The EIS studies suggested the presence of high conductivity, and CV revealed the existence of high electrocatalytic properties in the prepared composite material. Therefore, an LOD of 0.035 µM and a linear range of 0.5 to 100 µM were obtained under the optimized conditions. The proposed 4-NP sensor also demonstrated high selectivity, repeatability, reproducibility, and successful monitoring of 4-NP in wastewater samples. It was also reported, in another research study, that trimetallic MOF is a promising electrode material for the sensing of p-NP [75]. A NiCoMn (NCM)-based trimetallic MOF was fabricated and embedded with conductive polycaprolactone (PCL) nanofibers. The catalytic properties of the composite were evaluated by a machine learning approach to find the suitability and ideal composition of the composite material. The NCM/PCL-based electrode showed an LOD of 2.38 nM and high selectivity in the presence of interfering species. In another study [76], a novel sensor was fabricated by utilizing black phosphorus (BP)-functionalized covalent organic framework (COF) nanoflowers (NFs) as the sensing material. The fabricated electrode was capable of detecting p-NP in seawater samples. The BP/COF NFs-based electrode exhibited improved adsorption capacity for p-NP, which may be attributed to the presence of high surface area COF NFs, whereas the presence of BP nanosheets improved the conductivity of the COF NFs and stability via P-C bonds. The synergism in the prepared material and high carrier mobility of BP improved the detection of p-NP, and the fabricated sensor displayed an LOD of 0.03 µM and a linear range of 0.1 to 200 µM, with selectivity and real-sample recovery values. These promising properties and the sensing ability of the above-proposed sensor suggest its potential for real-time monitoring of p-NP in environmental samples.

**Figure 10:**
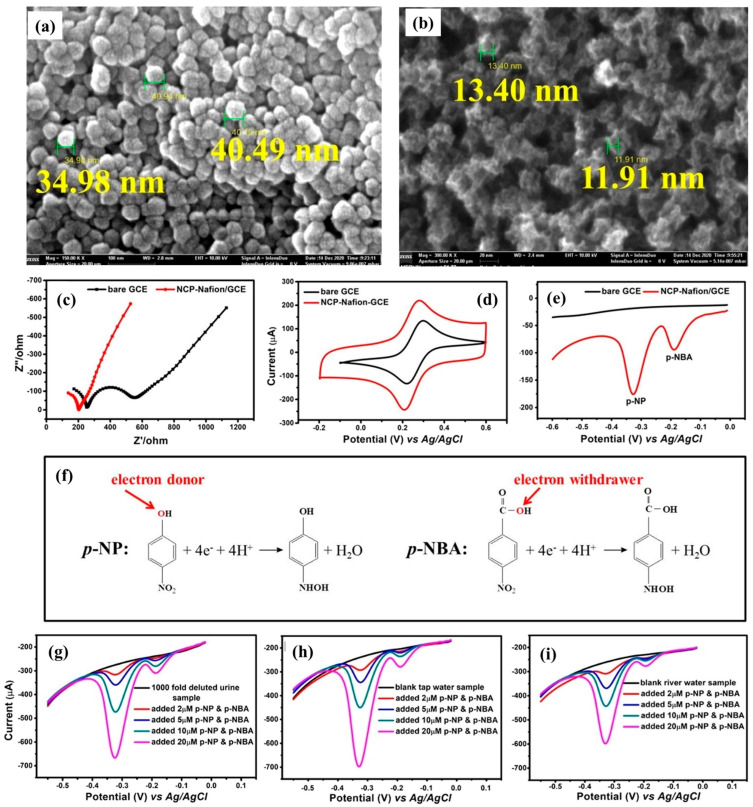
SEM image of (**a**) ZIF-8 and (**b**) NCP. (**c**) Nyquist plots and CV curves (**d**) of bare GCE and NCP-nafion/GCE in K_3_[Fe(CN)_6_]/K_4_[Fe(CN)_6_] redox system. (**e**) LSV curves of bare GCE and NCP-nafion/GCE in 10 µM p-NP and p-NBA. (**f**) Reaction mechanism for p-NP and p-NBA detection. LSV results for the monitoring of p-NP and p-NBA using NCP-nafion/GCE in human urine (**g**), tap water (**h**), and river water (**i**) samples. Reproduced with permission [77].

The zeolitic imidazolate frameworks (ZIFs) materials belong to the MOF family, which offers several promising features, such as high surface area, porous nature, abundant active sites, and reasonable catalytic properties. ZIF-derived materials also show good surface area and retained porous nature. In a previous study [77], Zn-based ZIF-8 was prepared using a benign synthesis approach at room temperature. Furthermore, ZIF-8-derived nanoporous carbon particles (NCP) were prepared. The synthesized materials were analyzed by a SEM technique, as shown in Figure 10a,b. The observations indicated that the prepared ZIF-8 and NCP have particle sizes of around 40 and 20 nm, respectively. The particle size for NCP was decreased due to the carbonization process. The authors also found that the prepared material had a high surface area of 1135.79 m^2^/g and pore size distribution of 3 to 5 nm. Furthermore, the prepared materials were deposited on a GCE surface for electrochemical sensing studies. The authors also used nafion as a binder to improve the stability of the catalyst on the electrode surface. Electrochemical impedance spectroscopy (EIS) was adopted to characterize the charge transfer kinetics of the bare GCE and NCP-nafion/GCE electrodes. The obtained results show that NCP-nafion/GCE has low charge transfer resistance (R_ct_), which suggests the presence of high conductivity compared to the bare GCE (Figure 10c). The CV studies also show that the constructed electrode using NCP as the electrode material possesses good electrocatalytic properties (Figure 10d). The LSV curves of the bare GCE and NCP-nafion/GCE for 10 µM p-NP and p-nitrobenzoic acid (p-NBA) are displayed in Figure 10e. The obtained results show that NCP-nafion/GCE has higher catalytic activity for the detection of p-NP and p-NBA compared to the bare GCE. The reaction mechanism for p-NP and p-NBA detection is shown in Figure 10f. The presence of the binder may improve the stability or adhesiveness on the electrode surface, but the conductivity of the material can be compromised. Therefore, despite the larger surface area of 1135.79 m^2^/g and nitrogen-rich NCPs, the modified electrode shows a linear range of 0.05 µM to 80 µM. We believe that the linear range could be better if the conductivity of the electrode was not compromised. Therefore, future studies should combine NCPs with other conductive and stable materials to form a stable composite material for the development of a p-NP sensor. The main advantage of this study was the efficient detection of p-NP in human urine, tap water, and river water samples with acceptable recovery, as shown in Figure 10g, Figure 10h, and Figure 10i, respectively.

In another research work [78], ZIF-8, SnO_2_, and gCN were combined to form a ternary composite for an electrochemical sensing application of p-NP. The electrochemical activities of the synthesized SnO_2_@ZIF-8/gCN composite-modified electrodes were examined using chronoamperometry, CV, and DPV techniques. The observations indicated that the SnO_2_@ZIF-8/gCN composite-modified electrode is a promising candidate for the monitoring of p-NP in environmental samples, with an LOD of 0.565 µM and sensitivity of 2.63 μA.cm^−2^.μM^−1^. This sensor also showed stability for 30 days and selective behavior for p-NP detection in the presence of various interfering species. Zhang et al. [79] reported the preparation of NiCo_2_S_4_ (NCS) on carbon paper (CP) using an electrodeposition method. Furthermore, Co-ZIF-L was grown on the surface of NCS to form the *n*-Co-ZIF-L/NCS-*X*/CP electrode. The fabricated electrode was found to be sensitive and selective for 4-NP detection and displayed an LOD of 0.134 µM and linear ranges of 0.5 µM to 100 µM and 100 µM to 1800 µM, with high sensitivity of 824.10 μA.mM^−1^·cm^−2^.

Huang et al. [80] proposed the fabrication of a novel composite of MXene and ZIF-derived materials for the fabrication of an electrochemical sensor. The authors prepared niobium carbide MXene (Nb_2_CTx) and Zn-Co-ZIF-derived bimetallic Zn, Co-embedded N-doped carbon (Zn-Co-NC) nanocages. The prepared composite (Nb_2_CTx/Zn-Co-NC) was characterized by XPS to confirm tits formation and to identify their valence states. The combined composite effects of Nb_2_CTx/Zn-Co-NC improved the electrochemical detection of 4-NP and showed a sensitivity of 4.65 μA.μM^−1^·cm^−2^, with a wide linear range of 1 μM to 500 μM, including an LOD of 0.070 μM. The fabricated electrode also displayed selectivity and stability, which may be ascribed to the high electrical conductivity, surface area of MXene, and electrostatic interactions between Nb_2_CTx and Zn-Co-NC. Gopi et al. [81] proposed a novel electrode material for the fabrication of electrochemical sensors for environmental monitoring. The authors prepared a silver bismuth sulphide (AgBiS_2_)-MXene composite and deposited it on a GCE surface, which was further explored for the monitoring of 4-NP in water samples. The DPV technique indicated that the MXene-AgBiS_2_-modified GCE showed a higher current response compared to the other electrodes, and the current response linearly increased with increasing 4-NP concentration. Therefore, the authors achieved an LOD of 0.00254 µM, an LOD of 0.02 µM to 1869 µM, and high sensitivity of 5.862 µA.µM^−1^·cm^−2^. The MXene-AgBiS_2_-modified GCE was also selective for the monitoring of 4-NP in the presence of interfering substances, including Co^2+^, glucose, Zn^2+^, urea, uric acid, Mn^2+^, Cu^2+^, dopamine, Na^+^, ascorbic acid, etc., which suggested its potential for practical applications.

**Figure 11:**
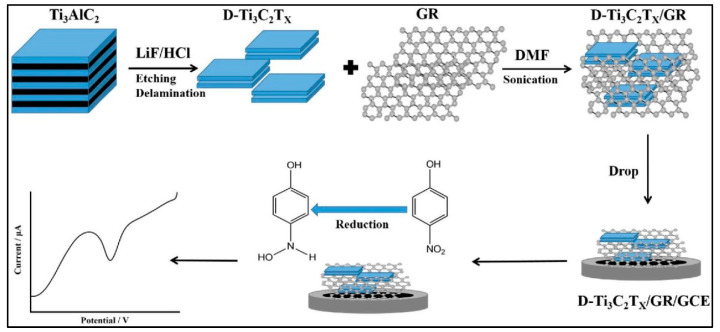
Schematic representation of the formation of D-Ti_3_C_2_T_x_/Gr composite and electrode modification for p-NP detection. Reproduced with permission [82].

Wang et al. [82] also adopted titanium carbide (Ti_3_C_2_T_x_) MXene-modified Gr as a promising composite material for the development of a p-NP electrochemical sensor (Figure 11). The synthesized delaminated Ti_3_C_2_T_x_/Gr composite-based electrode displayed high conductivity and low charge transfer resistance values, which suggested its potential for the detection of environmental pollutants such as p-NP. It can be stated that the Ti_3_C_2_T_x_/Gr composite-based electrode can be explored for real-time monitoring of p-NP due to its high reproducibility, stability, selectivity, and acceptable recovery in environmental samples.

In another study [83], coordination polymers (CPs: [ZnL_3_]*_n_*(X)_2*n*_, L = *trans*-1,4-bis(imidazolyl)-2-butene; X^−^ = BF_4_^−^, ClO_4_^−^, NO_3_^−^) were used for the monitoring of 4-NP, which exhibited reasonable electrochemical performance. This study suggested that coordination polymers can be used for electrochemical sensing applications. MXene/β-cyclodextrin (MXene/β-CD) was also used as a sensing material for the construction of an electrochemical sensor for the monitoring of o-NP, p-NP, and m-NP [84]. The MXene/β-CD-modified GCE delivered electrochemical performance for the sensing of o-NP, p-NP, and m-NP in terms of LOD, sensitivity, selectivity, and stability. The enhanced electrochemical performance of the MXene/β-CD-modified GCE may be ascribed to the electrochemically active sites, high surface area, and high electrical conductivity of the MXene/β-CD composite. MOFs, COFs ZIFs, and MXenes are promising electrode modifiers due to their surface area, porosity, conductive properties, and abundant active sites, which may facilitate the redox reactions at the modified electrode surface. The high surface area may improve the 4-NP adsorption, whereas the high conductivity of the electrode modifiers improves electron transfer and enhances the sensitivity of the constructed electrochemical sensors. Thus, fabrication of an MOF/MXene composite may be of particular significance for the development of 4-NP electrochemical sensors. The electrochemical performance of the above-mentioned sensors is summarized in Table 3.

### 2.4. Other Electrochemical Sensors

Trang et al. [85] used a novel electrode material for the detection of 4-NP in tomato samples. In this regard, the authors prepared Ag NPs using grapefruit peels, mangosteen peels, and natural plant extracts from green tea leaves. The observations revealed that green tea-based formation of the Ag NPs-modified electrode demonstrated high sensitivity and LOD due to the formation of crystalline Ag NPs without aggregation and uniform shape. Nehru et al. [86] prepared yolk-shell-structured molybdenum disulfide nanospheres (MoS_2_ NS) by employing a hydrothermal method. The prepared MoS_2_ NS showed good stability and sensitivity for the monitoring of 4-NP in river and tap water samples. The yolk-shell MoS_2_ NS-modified GCE displayed an LOD of 2.9 nM, a sensitivity of 1.2704 µA.µM^−1^·cm^−2^, and selectivity for 4-NP detection.

Poly(biphenol/biphenoquinone-vanadium (IV)) (poly BP/BQ-VIV) was synthesized using facile conditions [87]. The CPE was modified with poly BP/BQ-VIV, and an electrochemical analysis, i.e., DPV, approach indicated that the current response increased with increasing 4-NP concentration. An interesting LOD of 60 nM and sensitivity of 0.997 µA.µM^−1^ were obtained for 4-NP detection. In another study [88], electropolymerization of vitamin B complex (vitamin B_2_/riboflavin) on a pencil graphite electrode (PGE) was carried out for the monitoring of 4-NP. The poly-riboflavin (PRB)-modified PGE exhibited an LOD of 1.78 µM and a linear range of 5 µM to 700 µM. This study revealed that biosensors can be developed using PGE as an electrode for the detection of environmental pollutants. Bismuth dendrites were electrodeposited on a GCE surface for the detection of p-NP [89]. The improved conductivity and active sites of the modified electrode enhanced the selectivity and sensitivity of the 4-NP sensor. Silver amalgam particles (Ag Aps) were coated on a screen printed silver electrode (SPAgE) for the monitoring of 4-NP [90]. The optimized conditions revealed that the fabricated electrode delivered an LOD of 1 µM and high sensitivity. This electrode can be explored for the monitoring of environmental pollutants to detect toxic substances.

**Figure 12:**
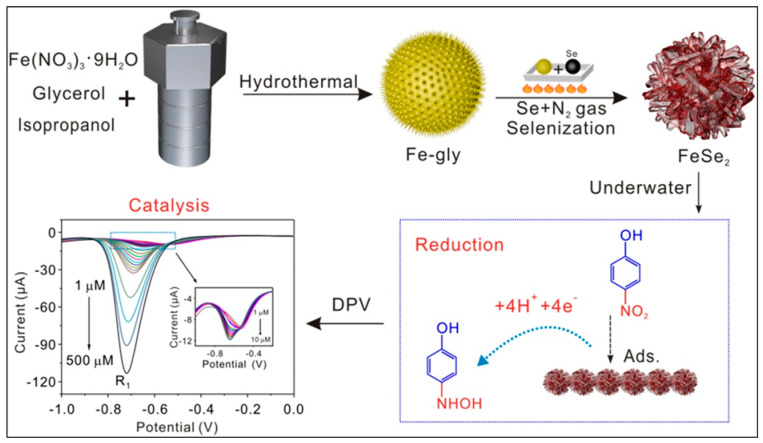
Schematic illustration of the preparation of FeSe_2_ and its role for p-NP detection. Reproduced with permission [92].

In another study [91], few-layer black phosphorus nanosheets (BPNS) were obtained by exfoliation of bulk BP in the presence of polyvinylpyrrolidone (PVP). It was observed that PVP improved the exfoliation and stability of BPNS. The few-layered BPNS-based material exhibited a high surface area, which improved the detection of 4-NP in terms of LOD, linear range, and real-sample recovery in wastewater samples. Iron selenide (FeSe_2_) with high electrical conductivity and a larger surface area was prepared using a hydrothermal method [92]. The preparation of the FeSe_2_ and its role for p-NP detection are displayed in Figure 12. The FeSe_2_-modified electrode exhibited acceptable stability and reproducibility. This improved performance may be attributed to the presence of conductivity, higher surface area, and catalytic behavior of the electrocatalyst.

Sajjan et al. [93] proposed the preparation of a cobalt (II) β-{4-[2-methoxy-4-(4-tolyimino-methyl)-phenoxy]} phthalocyanine complex incorporating a Schiff-base (CoTTIMPPc), which was characterized by various physicochemical techniques. The prepared CoTTIMPPc was coated on the GCE active area and used as an electrochemical sensor for the detection of 4-NP. The fabricated sensor exhibited an LOD of 38 nM and a linear range of 100 to 1000 nM using CV. The DPV analysis also indicated that the sensor could detect 4-NP with an LOD of 36 nM and a linear range of 100 to 1200 nM. Similarly, amperometric studies exhibited an LOD of 30 nM and a linear range of 100 to 800 nM. This indicates that all techniques show almost similar LOD. Thus, the electrode material plays a crucial role in the detection of 4-NP.

A novel lanthanum-based oxide (La_0.7_Sr_0.3_Cr_1−x_Ru_x_O_3_ (0 < x < 0.1)) was synthesized for the fabrication of an electrochemical sensor [94]. The optimized conditions revealed that La_0.7_Sr_0.3_Cr_0.925_Ru_0.075_O_3_ had improved catalytic activity for 4-NP detection. Therefore, the authors were able to achieve an LOD of 8 mM and a linear range of 25 to 5000 mM. Karvekar et al. [95] used pre-treated GCE (PGCE) as a working substrate for the fabrication of a 2-NP electrochemical sensor, which displayed promising electrochemical properties. Bao et al. [96] reported the preparation of biochar from biomass-derived lignin and cellulose through a pulping technique. Furthermore, it was used as a sensing layer for the construction of a 2-NP and 4-NP sensor. The proposed sensor displayed reasonable electrochemical performance for the detection of 2-NP and 4-NP in terms of LOD and sensitivity. Another research study revealed that 2-amino nicotinamide (2-AN) may be used as an electrode material to modify the surface of a GCE [97]. The 2-AN-modified GCE exhibited a higher current response, and the SWV analysis indicated that the current response linearly increased with increasing 2-NP amount. This proposed electrochemical sensor showed two wide linear ranges of 9.9 nM to 52.5 µM and 52.5 µM to 603 µM, with recoveries of 97.1 to 103.6% in tap/river water samples and an accepted standard deviation of 1 to 3.9%. Chavan et al. [98] reported the formation of N-doped biocarbons (AIBN_X_OC) by pyrolyzing orange peel waste with azobisisobutyronitrile (AIBN). The prepared material was sensitive for the detection of 4-NP, which displayed an LOD of 0.4 nM and selectivity. Anandkumar et al. [99] proposed the fabrication of a GCE with novel (CeGdHfPrZr)O_2_ (HEO) NPs for the detection of p-NP. It was found that the presence of HEO NPs on the GCE surface improved the electrocatalytic properties of the fabricated electrode for p-NP detection. The high sensitivity of 0.1517 μA.μM^−1^·cm^−2^ and satisfactory stability with high selectivity suggested its potential for environmental monitoring applications. Ag NPs were combined with whey protein amyloid fibrils (WPI-AFs) using facile conditions [100]. The Ag NPs/WPI-AFs were characterized by SEM and XRD, which confirmed the formation of the targeted material. The Ag NPs/WPI-Afs-based electrochemical sensor showed good selectivity, sensitivity, and LOD for p-NP detection. A novel FeC_x_-coated carbon sheet (FC) was also prepared using novel strategies [101]. This material was applied as the sensing material for the detection of 4-NP, which delivered an LOD of 84 nM and a sensitivity of 12.15 μA.μM^−1^·cm^−2^. This sensor also displayed selectivity for 4-NP detection in the presence of interfering species, such as 4-aminophenol, NaNO_3_, CaCl_2_, Na_2_SO_4_, CuCl_2_, resorcinol, 2-aminophenol, hydroquinone, and catechol. Malvessi et al. [102] fabricated SPE-based electrochemical sensors using carbon-based ink as the electrode material. The ink was prepared using nanographite, graphene nanoplates, and alkyd resin (wt ratio = 40:10:50%). The SWV analysis suggested that the current response for 4-NP detection increased with respect to the concentration of 4-NP. Therefore, an LOD of 2.8 µM was obtained for p-NP detection under the optimized conditions.

In another study [103], Co-based conjugated coordination polymers (CoCCPs) were prepared by using planar π-conjugated 2, 5-diamino-1, 4-benzenedithiol as an organic ligand. The CoCCPs-modified electrode exhibited an LOD of 0.00986 µM for the detection of p-NP. This electrode was also efficient for the monitoring of p-NP in wastewater samples. A two-step synthesis method was reported for the preparation of a Ni_x_ NPs/mesoporous carbon (MPC) composite under facile conditions [104]. The Ni wt % was varied in the range of 10 to 20%, and the authors found that 15 wt % Ni is sufficient for the electrochemical detection of p-NP using the DPV technique. The improved performance may be ascribed to the higher surface area and faster electron transfer, active sites, and high electrical conductivity. Therefore, the authors achieved an LOD of 1.8 µM and a broad linear range of 170 µM to 10,000 µM, with good stability. A molecularly imprinted polymer (MIP)-based electrochemical sensor was also reported for the detection of p-NP [105]. The proposed MIP was synthesized using facile conditions, and its electrochemical activity for p-NP was evaluated, which displayed an LOD of 0.2 µM and good recovery in wastewater samples. Gopi et al. [106] fabricated a novel 4-NP electrochemical sensor using cerium-iron phosphate (CeFeP)-modified GCE. The authors found that TEM studies revealed the presence of a flower-like structure of CeFeP. The flower-like structure comprised uniform rods. The CeFeP-modified electrode showed good electrocatalytic activity for the reduction of 4-NP and electrochemical detection of 4-NP. The obtained results were found to be satisfactory in terms of a wide linear range of 0.1 to 50 µM and an LOD of 0.01 µM. There are some reports that exist for the detection of 4-NP and other isomers and derivatives using electrochemical sensors [107,108,109,110,111,112]. The electrochemical sensing performance of the previously reported sensors is shown in Table 4.

It is observed from the above-discussed reported literature that the SWV technique is more effective for the detection of 4-NP using nanostructured material-modified electrodes. The gCN/V_2_O_5_-modified GCE delivered an LOD of 0.00085 µM [22]. The L-Cys/Nd_2_O_3_/rGO/GCE also exhibited an LOD of 0.02 µM for the monitoring of 4-NP using the SWV method [64]. Furthermore, it is also observed that GO/CNF displayed a low LOD value of 0.0008 µM for 4-NP detection using the DPV technique [29]. The CSAC/SrSnO_3_/PPy-based DPV investigations demonstrated an LOD of 0.00015 µM for the quantification of 4-NP [67]. Similarly, DPV-based studies revealed that Mxene-AgBiS_2_/GCE is highly sensitive for 4-NP detection and demonstrated an LOD of 0.00254 µM [81]. We believe that SWV and DPV are both promising techniques for the detection of 4-NP, as the summarized results in reference number [22,29] are almost similar, whereas the electrode materials are different. Thus, it can be concluded that SWV and DPV are promising techniques for the quantification of 4-NP. The DPV and SWV approaches can be used for the detection of 4-NP with low LOD values. However, it would be worthy to mention here that DPV- and SWV-based sensors may suffer from selectivity issues due to the existence of 4-NP isomers. The SWV and DPV techniques may exhibit low selectivity for 4-NP detection in the presence of the isomeric mixture (m-NP or o-NP). On the other hand, the Amp technique also exhibits an interesting LOD value of 0.01 µM for the detection of 4-NP using BVG@C [23]. The Amp technique is a well-known approach for the selective detection of various analytes. In our opinion, we would like to state that the DPV or SWV approach is a suitable method for the detection of 4-NP in the absence of the isomeric mixtures, whereas the Amp technique can be used for the selective detection of 4-NP.

## 3. Conclusions, Challenges, and Future Directions

It may be worthy to conclude that various electrode materials, such as carbon-based materials, metal oxides, polymers, MXenes, MOF, and other hybrid composite materials, have been extensively used for the fabrication of electrochemical sensors for 4-NP detection. It was observed that gCN/V_2_O_5_/GCE exhibits an LOD of 0.00085 µM, which may be attributed to better electron transportation and synergistic interactions. It was also observed that g-gCN/V_2_O_5_/GCE can be used to detect 4-NP, with a wide linear range of 0.001 to 100 µM through the SWV technique. Similarly, a GO/CNF-based electrochemical sensor displayed a low LOD of 0.0008 µM for p-NP detection using the DPV technique. This improved sensing performance of the proposed GO/CNF-based sensor may be ascribed to the presence of active sites and defects on the GO surface. The CSAC/SrSnO_3_/PPy-based electrode also displayed an LOD of 0.00015 µM for 4-NP detection. The Ni@CuO/rGO/PtE displayed an LOD of 0.0054 µM for the detection of 4-NP using the DPV approach. In contrast, Mxene-AgBiS_2_/GCE demonstrated an LOD of 0.00254 µM for the detection of 4-NP through DPV analysis. CoCCPs-1 also displayed a low LOD of 0.00986 µM for p-NP detection. The reported literature shows that electrochemical sensors have selectivity for the detection of 4-MP. However, it should be noted that isomers of 4-NP, such as o-NP or m-NP, affect the selectivity due to the nearly or almost identical redox active nitro and phenolic functionalities. Therefore, challenges exist during the detection of 4-NP in presence of o-NP or m-NP. However, simultaneous detection of 4-NP, o-NP, and m-NP is possible, but it would be difficult to identify 4-NP in the isomeric mixture. Thus, we believe that future studies should focus and report the interference tolerance and peak separation potential values. In addition, selectivity for 4-NP detection can be enhanced by defect engineering and surface functionalization approaches. It can be observed that the DPV technique may be one of the promising approaches for the detection of p-NP/m-NP/o-NP and other isomers/derivatives of the nitrophenol family. It is also observed that hybrid composites exhibit synergistic interactions and improved conductive properties, which facilitate the redox reactions at the electrode surface and enhance the sensitivity of the electrochemical sensors. The electrochemical sensors are also promising candidates for environmental monitoring applications due to their high selectivity, sensitivity, stability, repeatability, and reproducibility. Despite the significant progress in the detection of 4-NP using various advanced electrode materials, several challenges exist, which are mentioned below.

1. The DPV technique is a simple and promising electrochemical approach for the detection of nitrophenol compounds, but the presence of isomers may affect the selectivity. Thus, some novel strategies need to be developed to overcome this issue.
2. The exact mechanism for the detection of 4-NP at the electrode surface is not clear. It needs to be studied in detail.
3. Interface engineering using composite materials as electrode materials needs to be improved to enhance the sensitivity of electrochemical sensors.
4. Metal oxides exhibit good stability, but the presence of low conductivity is one of the major concerns.
5. Polymers exhibit high conductivity, but their long-term stability may restrict their potential for practical applications.
6. A new class of 2D-layered materials such as MXene displayed high conductivity and catalytic activity for electrochemical applications, but the synthesis methods, such as acid etching treatments, are not environmentally friendly.

Future studies may consider the following points for the development of electrochemical sensors for environmental monitoring applications.

i. Environmentally friendly synthesis methods should be developed for the preparation of MXenes.
ii. MXenes/layered double hydroxide (LDH)-based composite materials need to be optimized for the construction of electrochemical sensors.
iii. Electrochemical sensors can be integrated with flexible and wearable devices for environmental monitoring applications.

## Figures and Tables

**Table 1:** Electrochemical performance of the reported sensors.

Electrode Material	Analyte	Technique	LOD (µM)	Linear Range (µM)	Sensitivity	Ref.
gCN-PD/GCE	4-NP	DPV	0.039	2.854 to 1117	0.213 μA.μM^−1^	[21]
gCN/V_2_O_5_/GCE	4-NP	SWV	0.00085	0.001 to 100	-	[22]
BVG@C	4-NP	Amp	0.01	10 to 350	2.368 μA.mM^−1^·cm^−2^	[23]
gCN@CeO_2_/CdO/Nafion/GCE	4-NP	LSV	0.45	3 to 8	0.365 µA.µM^−1^·cm^−2^	[24]
MoS_2_/S-gCN	4-NP	DPV	0.0128	0.1 to 2.6	-	[25]
gCN-MoS_2_-Au	4-NP	LSV	0.2192	0 to 1000	-	[26]
Gr-Ag/PPE	4-NP	CV	2.70	3.125 to 100	-	[27]
Au-rGO/AC/GCE	4-NP	LSV	1.12	72 to 4400	0.01108 µA.µM^−1^	[28]
Au-rGO/AC/GCE	4-NP	Amp	0.36	18 to 592	0.03436 μA.µM^−1^	[28]
GO/CNF	p-NP	DPV	0.0008	0.003 to 210	-	[29]
P(Arg)/eG/SPE	p-NP	SWV	0.012	0.5 to 1250	0.661 μA.μM^−1^·cm^−2^	[30]
rGO-MoS_2_/Fe_3_O_4_	p-NP	DPV	0.8	10 to 400	0.71 μA.μM^−1^·cm^−2^	[31]
Ni_3_Se_4_/rGO/ITO	4-NP	DPV	0.0171	0.05 to 5 and 5 to 200	-	[33]
Pd-His-GQD-G/PNIPAM	4-NP	DPV	0.10	0.5 to 250	-	[35]
NdV/MWCNTs-OH	p-NP	LSV	0.041	1 to 200	-	[37]
PEI-MWCNTs-COOH/GCE	p-NP	SDLSV	0.04	0.2 to 100	1.5212 μA.μM^−1^	[38]
MWCNT@Hg/YO NCs/Nafion/GCE	4-NP	LSV	0.196	0.01 to 23.0	6.233 μA.μM^−1^·cm^−2^	[40]
CMS/GCE	p-NP	DPV	0.2	2 to 1000	-	[43]
OLC/GCE	4-NP	LSV	0.00374	0.05 to 120	-	[44]
CalCOP-MPC/GCE	p-NP	DPV	0.212	1 to 400	-	[45]
GCE/CNF/CuCrO_2_	4-NP	DPV	0.022	0.1 to 150	20.02 μA.μM^−1^·cm^−2^	[47]

**Table 2:** Electrochemical performance of the reported sensors.

Electrode Material	Analyte	Technique	LOD (µM)	Linear Range (µM)	Sensitivity	Ref.
Ag_2_O-ZnO CNCs/Au	4-NP	DPV	0.023	0.4 to 26 and 28 to 326	1.6 µA.µM^−1^·cm^−2^	[49]
CPE-α-Fe_2_O_3_ @Mg/Al- CO_3_-LDH	p-NP	SWV	4.98	-	-	[50]
Ag_2_O NPs/Au	4-NT	LSV	0.0623	0.6 to 5.9 μM and 37 to 175 μM	-	[51]
CuBi_2_O_4_	4-NP	Chronoamperometry	0.61	-	56.16 μA.μM^−1^·cm^−2^	[52]
Fe_3_O_4_@Fe-BTC/SPE	4-NP	DPV	0.49	0.1 to 50	-	[54]
Au electrode/Sm_2_O_3_ NPs	p-NP	CV	0.5033	0.1 to 7	-	[56]
CeVO_4_	p-NP	SWV	0.091	0.2 to 60	-	[57]
Y@SnO_2_-ZnO/nafion/GCE	2-NP	LSV	1.3885	0.1 to 7.5	120.25 µA.μM^−1^·cm^−2^	[59]
Mn-Fe_3_O_4/_3D-G (M-C)	4-NP	DPV	0.019	5 to 100	-	[61]
V_o_-S-WO_2.9_/gCN/GCE	4-NP	DPV	0.133	0.4 to 100	-	[62]
L-Cys/Nd_2_O_3_/rGO/GCE	p-NP	SWV	0.02	-	-	[64]
CSAC/SrSnO_3_/PPy	4-NP	DPV/I-V	0.00015	1.0 nM to 10.0 mM	6.329 µA.mM^−1^·cm^−2^	[67]
rGO	4-NP	DPV	4.2	50 to 800	-	[68]
Ni@CuO/rGO/PtE	4-NP	DPV	0.0054	0.09 to 105	-	[69]
ZnCo_2_O_4_/MWCNTs/GCE	4-NP	CV	0.026	0.5 to 600	-	[70]
CeO_2_@C-dots nanosphere	4-NP	CV	0.94	-	3.4 μA.μM^−1^·cm^−2^	[71]

**Table 3:** Electrochemical performance of the reported sensors.

Electrode Material	Analyte	Technique	LOD (µM)	Linear Range (µM)	Sensitivity	References
Mn-MOF@rGO	p-NP	DPV	0.078	0.75 to 140	-	[72]
Mn-MOF@rGO	o-NP	DPV	0.080	0.5 to 180	-	[72]
CPE/Cu-BTC	4-NP	DPV	0.040	0.7 to 10 and 10 to 100	-	[73]
CPE/Cu-Pic	4-NP	DPV	0.09	1.2 to 70	-	[73]
Cu-MOF/NGO/SPCE	4-NP	DPV	0.035	0.5 to 100	0.45 µA.µM^−1^·cm^−2^	[74]
BP/COF-NFs/GCE	p-NP	SWV	0.03	0.1 to 200	-	[76]
NCP-Nafion/GCE	p-NP	LSV	0.017	0.05 to 80	-	[77]
SnO_2_@ZIF-8/gCN	p-NP	DPV	0.565	10 to 100	2.63 μA.cm^−2^.μM^−1^	[78]
Nb_2_CT_X_/Zn-Co-NC	4-NP	DPV	0.070	1 to 500	4.65 μA.μM^−1^·cm^−2^	[80]
Mxene-AgBiS_2_/GCE	4-NP	DPV	0.00254	0.02 to 5 and 10 to 78	-	[81]
D-Ti_3_C_2_T_X_/GR	p-NP	DPV	0.16	1 to 175	-	[82]
MXene/*β*-CD/GCE	p-NP	DPV	0.042	0.5 to 180	-	[84]
MXene/*β*-CD/GCE	o-NP	DPV	0.063	0.4 to 300	-	[84]
MXene/*β*-CD/GCE	m-NP	DPV	0.087	0.5 to 300	-	[84]

**Table 4:** Electrochemical performance of the previously reported sensors.

Electrode Material	Analyte	Technique	LOD (µM)	Linear Range (µM)	Sensitivity	Ref.
GT-AgNPs/SPE	4-NP	DPV	0.43	0.5 to 50	1.25 μA.μM^−1^·cm^−2^	[85]
Yolk-shell MoS_2_ NS/GCE	4-NP	DPV	0.0029	0.005 to 717.205	-	[86]
Porous MoS_2_ NS/GCE	4-NP	DPV	0.022	0.005 to 412.205	-	[86]
Poly BP/BQ–V^IV^	4-NP	DPV	0.006	0.05 to 200	0.997 µA.µM^−1^	[87]
A-GCE/Bi	p-NP	DPV	0.00018	0.005 to 1.6 and 1.6 to 170	29.4 μA.μM^−1^·cm^−2^	[89]
PVP@BPNS	p-NP	DPV	0.028	0.10 to 5.0	-	[91]
GCE/CoTTIMPPc	4-NP	DPV	0.036	0.1 to 1.2	-	[93]
LPC-800/GCE	4-NP	DPV	0.0174	1 to 100	-	[96]
CPC-900/GCE	2-NP	DPV	0.0203	1 to 100	-	[96]
2-AN/GC	2-NP	SWV	0.00292	0.0099 to 52.5 and 52.5 to 603	-	[97]
(CeGdHfPrZr)O_2_/GCE	p-NP	SWV	0.32	5 to 100	-	[99]
AgNP-WPI-AF/SPCE	p-NP	DPV	0.963	40 to 118.6	0.592 μA.μM^−1^·cm^−2^	[100]
CoCCPs-1	p-NP	DPV	0.00986	0.05 to 5	-	[103]
MIP GCE	p-NP	CV	0.2	2 to 400	-	[105]
Fe-MIL-101	o-NP	DPV	0.013	0.036 to 86.4	-	[107]
CoC@Mn	o-NP	SWV	0.16	0.5 to 100	-	[108]
Au NPs@ZnO/CTSN NCs	2-NP	LSV	0.45	15 to 150	20.99 μA.μM^−1^·cm^−2^	[109]
COF/NH_2_-CNTs	o-NP	LSV	0.03	0.1 to 100 and 100 to 1000	8.66 and 1.44 μA.μM^−1^·cm^−2^	[110]
ZnO-CuS NHS 2/Au	4-NT	DPV	77.5 nM	0.769 to 38.00	1.87 μA.μM^−1^·cm^−2^	[111]
ZnS/f-CNF@PDA	3-NP	DPV	0.85	0.05 to 497	0.12 μA.μM^−1^·cm^−2^	[112]

## Data Availability

No new data were generated. The authors are unable to provide data.
